# Preoperative CT texture features predict prognosis after curative resection in pancreatic cancer

**DOI:** 10.1038/s41598-019-53831-w

**Published:** 2019-11-22

**Authors:** Hyung Sun Kim, Young Jae Kim, Kwang Gi Kim, Joon Seong Park

**Affiliations:** 1 Pancreatobiliary Cancer Clinic, Department of Surgery, Gangnam Severance Hospital, Yonsei University, Seoul, Korea; 2 Department of Biomedical Engineering, Gachon University College of Medicine, Incheon, Korea

**Keywords:** Pancreatic cancer, Predictive markers

## Abstract

Pancreatic cancer is a lethal disease, and resistance to chemotherapy is a critical factor influencing the postoperative prognosis. Tumour heterogeneity is an important indicator of chemoresistance. Therefore, we analysed tumour heterogeneity in preoperative computed tomography scans by performing texture analysis using the grey-level run-length matrix and analysed the correlation of survival with the value obtained in these analyses. We analysed 116 consecutive patients who underwent curative resection and had preoperative contrast-enhanced computed tomography data available for analysis. A region of interest was drawn on all slices with a visible tumour and normal pancreas on the arterial phase computed tomography scans; the correlation of pathological characteristics with grey-level run-length matrix features was analysed. We then performed Kaplan–Meier survival curve analysis among pancreatic cancer patients. The grey-level non-uniformity values in grey-level run-length matrix features for tumours were higher than those for normal pancreas. High grey-level non-uniformity values represent a non-uniform texture, i.e., heterogeneity. Grey-level run-length matrix features showed that recurrence-free survival was shorter in the group with high grey-level non-uniformity 135 values (p = 0.025). Our analyses of the correlation between pathological outcomes and grey-level run-length matrix features in pancreatic cancer patients showed that grey-level non-uniformity values were powerful prognostic indicators.

## Introduction

Pancreatic cancer is a lethal disease with 2-year metastasis and recurrence rates of 80% even in patients who undergo curative resection because of resistance to chemotherapy^1,2^. Chemoresistance is an important factor influencing the prognosis after curative resection, and tumour heterogeneity is an important indicator of the chemoresistance. Although other studies are underway to predict the prognosis or to characterise cancer phenotypes^3,4^, image texture analysis is currently used to predict tumour heterogeneity. In this regard, several studies have reported the correlation between tumour heterogeneity and the prognosis in oesophageal cancer, lung cancer, and cervical cancer^5–8^. However, only a few studies have reported texture analyses of tumour heterogeneity in pancreatic cancer^9^.

Texture analyses employ several methods, such as grey-level co-occurrence matrix (GLCM) and histogram analyses, to calculate or classify objects based on their texture. By assessing the distribution of grey levels, coarseness, and regularity within a lesion, computed image analysis algorithms can provide additional morphological information related to tumour heterogeneity. These texture analyses also offer the advantage of quantifying tumour heterogeneity, which cannot be reliably achieved by simple visual analysis. Therefore, in this study, we analysed tumour heterogeneity using preoperative computed tomography (CT) by performing texture analysis with the grey-level run-length matrix (GLRLM) approach. We then used these data to analyse the correlation between survival and the obtained tumour heterogeneity values in a large sample of curative-intent resected pancreatic cancers.

## Results

### Clinical characteristics of the patients with pancreatic cancer

Table 1 shows the patient characteristics in this study. The median age of the patients was 65.0 years, and the study population included 56 men (48.3%). In the T stage classification (American Joint Committee on Cancer (AJCC) 8^th^ edition^10^), 21 (18.1%) patients had T1 stage disease; 77 patients (66.4%), T2 stage disease; and 18 (15.5%) patients, T3 stage disease. In the N stage classification (AJCC 8^th^ edition), 42 (36.2%) patients had N0 stage disease; 55 (47.4%) patients, N1 stage disease; and 19 (16.4%) patients, N2 stage disease. Perineural invasion was observed in 98 patients (84.5%), while lymphovascular invasion was observed in about half of the patients (n = 57, 49.1%). Most patients showed moderate differentiation (n = 92, 79.3%)

**Table 1:** Pathological characteristics in pancreatic cancer patients.

Age		Median [Q1–Q3] or N (%)
		65.0 [55.75–70.25]
Sex	Men	56 (48.3%)
	Women	60 (51.7%)
T stage (8^th^)	T1	21(18.1%)
	T2	77 (66.4%)
	T3	18 (15.5%)
N stage (8^th^)	N0	42 (36.2%)
	N1	55 (47.4%)
	N2	19 (16.4%)
Stage (8^th^)	IA	12 (10.3%)
	IB	22 (18.9%)
	IIA	8 (7%)
	IIB	55 (47.4%)
	III	19 (16.4%)
PNI	Positive	98 (84.5%)
	Negative	17 (14.6%)
	Unknown	1 (0.9%)
LVI	Positive	57 (49.1%)
	Negative	58 (50%)
	Unknown	1 (0.9%)
Differentiation	WD	10 (8.6%)
	MD	92 (79.3%)
	PD	13 (11.2%)
	Unknown	1 (0.9%)

PNI; Perineural invasion, LVI; Lymphovascular invasion, WD; Well differentiated, MD; Moderate differentiated, PD; Poorly differentiated.

### GLRLM features and their correlation with pathological characteristics

Texture analysis was performed using ROIs (region of interests) of the same size in the arterial phase images of preoperative CT scans. The GLN(Grey-level non-uniformity) values for GLRLM features in the tumour were higher than those for the normal pancreas (median value of GLN0, GLN45, and GLN90 in normal tissues = 0.0543; GLN135 in normal tissues = 0.055; median value of GLN0, GLN45, and GLN90 in the tumour = 0.0553; GLN135 in the tumour = 0.0563).

Tables 2 and 3 show the results for the GLN features. Table 2 shows the results for GLN0, GLN45, and GLN90, indicating significant differences in the T stage. The proportion of cases with stage T3 disease was higher in the group with values higher than the median than in the group with values lower than the median (p = 0.046). The proportion of poorly differentiated tumours was higher in the group with values higher than the median (p = 0.054). For GLN135, there were no significant factors except the T stage and differentiation (Table 3). The proportion of cases with stage T3 disease and poorly differentiated tumours was higher in the group with values higher than median.

**Table 2:** Pathological characteristics and the value of GLN 0,45,90 in pancreatic cancer patients.

		GLN 0,45,90		p-value
		Value < Median (n = 48)	Value > Median (n = 68)	
Age	≤65	28 (58.3%)	31 (45.6%)	0.176
	>65	20 (41.7%)	37 (54.4%)	
Sex	Men	26 (54.2%)	30 (44.1%)	0.286
	Women	22 (45.8%)	38 (55.9%)	
T stage (8^th^)	T1	6 (12.5%)	15 (22.1%)	0.046
	T2	38 (79.2%)	39 (57.4%)	
	T3	4 (8.3%)	14 (20.6%)	
N stage (8^th^)	N0	16 (33.3%)	26 (38.2%)	0.543
	N1	22 (45.8%)	33 (48.5%)	
	N2	10 (20.8%)	9 (13.2%)	
Stage (8^th^)	IA	4 (8.3%)	8 (11.8%)	0.664
	IB	10 (20.8%)	12 (17.6%)	
	IIA	2 (4.2%)	6 (8.8%)	
	IIB	22 (45.8%)	33 (48.5%)	
	III	10 (20.8%)	9 (13.2%)	
PNI	Positive	42 (87.5%)	56 (82.4%)	0.590
	Negative	6 (12.5%)	11 (16.2%)	
	Unknown	0 (0%)	1 (1.5%)	
LVI	Positive	21 (43.8%)	36 (52.9%)	0.401
	Negative	27 (56.3%)	31 (45.6%)	
	Unknown	0 (0%)	1 (1.5%)	
Differentiation	WD	8 (16.7%)	2 (2.9%)	0.054
	MD	36 (75%)	56 (82.4%)	
	PD	4 (8.3%)	9 (13.2%)	
	Unknown	0 (0%)	1 (1.5%)	

PNI; Perineural invasion, LVI; Lymphovascular invasion, WD; Well differentiated, MD; Moderate differentiated, PD; Poorly differentiated.

**Table 3:** Pathological characteristics and the value of GLN 135 in pancreatic cancer patients.

		GLN 135		p-value
		Value < Median (n = 50)	Value > Median (n = 66)	
Age	≤65	28 (56%)	31 (47%)	0.335
	>65	22 (44%)	35 (53%)	
Sex	Men	27 (54%)	29 (43.9%)	0.283
	Women	23 (46%)	37 (56.1%)	
T stage (8^th^)	T1	7 (14%)	14 (21.2%)	0.055
	T2	39 (78%)	38 (57.6%)	
	T3	4 (8%)	14(21.2%)	
N stage (8^th^)	N0	17 (34%)	25 (37.9%)	0.363
	N1	22 (44%)	33 (50%)	
	N2	11 (22%)	8 (12.1%)	
Stage (8^th^)	IA	5 (10%)	7 (10.6%)	0.551
	IB	10 (20%)	12 (18.2%)	
	IIA	2 (4%)	6 (9.1%)	
	IIB	22 (44%)	33 (50%)	
	III	11 (22%)	8 (12.1%)	
PNI	Positive	44 (88%)	54 (81.8%)	0.520
	Negative	6 (12%)	11 (16.7%)	
	Unknown	0 (0%)	1 (1.5%)	
LVI	Positive	22 (44%)	35 (53%)	0.394
	Negative	28 (56%)	30 (45.5%)	
	Unknown	0 (0%)	1 (1.5%)	
Differentiation	WD	8 (16%)	2 (3%)	0.081
	MD	37 (74%)	55 (83.3%)	
	PD	5 (10%)	8 (12.1%)	
	Unknown	0 (0%)	1 (1.5%)	

PNI; Perineural invasion, LVI; Lymphovascular invasion, WD; Well differentiated, MD; Moderate differentiated, PD; Poorly differentiated.

### Survival analysis

**Figure 1:**
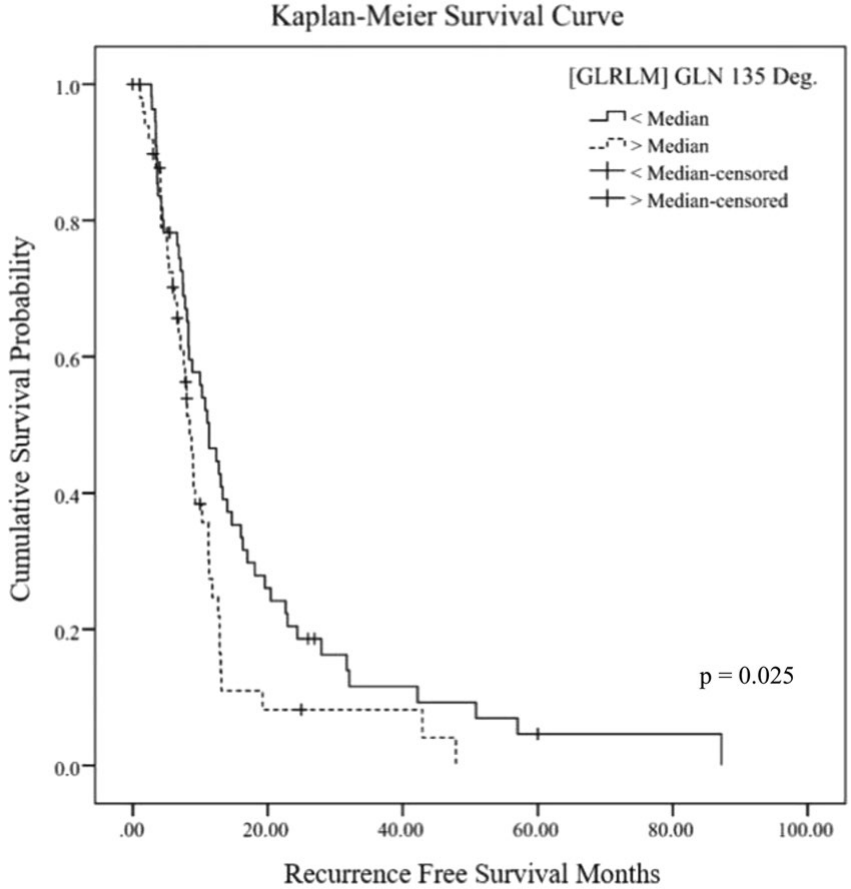
Kaplan Meier survival curve for recurrence-free survival according to the GLN feature (GLN135).

The GLN values were used to create a Kaplan–Meier survival curve. Recurrence-free survival was shorter in the group with high GLN135 values (p = 0.025) (median recurrence-free survival: group with values higher than the median = 6.72 months, group with values lower than the median = 10.52 months). As described above, GLN indicates heterogeneity. This result showed that a high GLN135 value was associated with a poor prognosis (Fig. 1).

### Prognostic impact of clinicopathologic features in pancreatic cancer

On multivariate analyses, node stage, tumour differentiation, and GLN135 value were identified as independent factors for poor recurrence-free survival (Table 4).

**Table 4:** Univariate and multivariate analyses of the relationship between RFS and clinicopathologic variables by Cox regression hazard model.

Factors	Univariate analysis			Multivariate analysis		
	HR	95% CI	*P*	HR	95% CI	*P*
**Age**						
≤65	1			1		
>65	1.453	0.955–2.211	0.081	1.548	0.996–2.405	0.052
**Sex**						
Men	1					
Women	0.920	0.607–1.392	0.692			
**T stage (8th)**						
T1	1					
T2	1.316	0.723–2.398	0.369			
T3	1.891	0.864–4.139	0.111			
**N stage (8**^**th**^**)**						
N0	1			1		
N1	1.529	0.972–2.405	0.066	1.623	1.014–2.598	**0.043**
N2	1.574	0.784–3.158	0.202	2.804	1.319–5.957	**0.007**
**Stage (8**^**th**^**)**						
**IA**	1					
**IB**	0.987	0.443–2.199	0.974			
**IIA**	1.641	0.538–5.007	0.384			
**IIB**	1.619	0.781–3.354	0.195			
**III**	1.676	0.678–4.146	0.264			
**PNI**						
Positive	1					
Negative	0.851	0.470–1.541	0.594			
**LVI**						
Positive	1					
Negative	0.736	0.484–1.120	0.153			
**Differentiation**						
WD	1			1		
MD	2.238	1.071–4.676	**0.032**	2.309	1.072–4.976	**0.033**
PD	2.906	1.160–7.282	**0.023**	3.290	1.207–8.969	**0.020**
**GLN0, 45, 90**						
Value < median	1			1		
Value > median	1.476	0.967–2.254	0.071	0.254	0.056–1.150	0.075
**GLN135**						
Value < median	1			1		
Value > median	1.696	1.102–2.610	**0.016**	6.030	1.317–27.596	**0.021**

PNI; Perineural invasion, LVI; Lymphovascular invasion, WD; Well differentiated, MD; Moderate differentiated, PD; Poorly differentiated, RFS; Recurrence free survival.

### Prognostic impact of clinicopathologic features in pancreatic cancer

On multivariate analyses, tumour stage, tumour differentiation, and perineural invasion were identified as independent factors for poor overall survival (Table 5).

**Table 5:** Univariate and multivariate analyses of the relationship between OS and clinicopathologic variables by Cox regression hazard model.

Factors	Univariate analysis			Multivariate analysis		
	HR	95% CI	*P*	HR	95% CI	*P*
**Age**						
≤65	1					
>65	1.402	0.931–2.112	0.106			
**Sex**						
Men	1					
Women	0.741	0.493–1.115	0.151			
**T stage (8th)**						
T1	1					
T2	1.321	0.748–2.332	0.337			
T3	2.195	1.086–4.435	**0.028**			
**N stage (8**^**th**^**)**						
N0	1					
N1	1.561	0.983–2.477	0.059			
N2	2.011	1.086–3.726	**0.026**			
**Stage (8**^**th**^**)**						
**IA**	1			1		
**IB**	1.094	0.457–2.619	0.840	1.133	0.472–2.720	0.780
**IIA**	2.169	0.776–6.064	0.140	2.397	0.832–6.909	0.105
**IIB**	1.888	0.890–4.006	0.098	2.346	1.094–5.029	**0.028**
**III**	2.449	1.035–5.794	**0.041**	3.098	1.298–7.390	**0.011**
**PNI**						
Positive	1			1		
Negative	0.542	0.279–1.052	0.070	0.450	0.221–0.916	**0.028**
**LVI**						
Positive	1					
Negative	0.714	0.474–1.075	0.107			
**Differentiation**						
WD	1			1		
MD	1.493	0.744–2.996	0.259	1.686	0.834–3.407	0.146
PD	1.830	0.741–4.520	0.190	3.943	1.523–10.207	**0.005**
**GLN0, 45, 90**						
Value < median	1					
Value > median	1.129	0.747–1.705	0.565			
**GLN135**						
Value < median	1					
Value > median	1.135	0.753–1.711	0.546			

PNI; Perineural invasion, LVI; Lymphovascular invasion, WD; Well differentiated, MD; Moderate differentiated, PD; Poorly differentiated, OS; Overall survival.

## Discussion

Quantitative imaging techniques based on radiomics have recently gained prominence as potential methods to provide mineable data from imaging analysis using automatically extracted data-characterisation processes^11–14^. CT is a good technique for assessment of the structural aspects of tumours and the normal adjacent organs. It remains the initial imaging method used for clinical staging of pancreatic cancer and for evaluating local spread into adjacent structures.

Tumour heterogeneity is a significant prognostic factor because it reflects the subclonal populations of the tumour. Tumours show temporally and spatially heterogeneous features in imaging studies (CT, MRI, and PET-CT). Since CT reflects the characteristics of these features of tumours, studies on the evaluation of tumour heterogeneity using preoperative CT scans are underway for various tumours^15–22^. Therefore, we used CT texture analysis to determine the predictive value of preoperative CT scans in pancreatic cancer.

In this study, we analysed the differences in GLRLM values between normal pancreatic tissue and the tumours in pancreatic cancer. The GLN values were higher than normal in tumours. High GLN values represent a non-uniform texture, i.e., heterogeneity. The group with higher GLN values also showed poorer results for prognostic factors such as T stage and differentiation type. In this study, tumour heterogeneity was reflected by a high GLN value in the image and was associated with increased biological aggressiveness (higher T stage and poor differentiation of the tumour).

The biological rationale for radiologic features of heterogeneity is to determine potential histopathologic correlates (tumour grade, hypoxia, and angiogenesis-specific genetic and molecular features). Higher tumour heterogeneity, as reflected by lower uniformity of positive pixel values and greater variability in texture analysis, correlates with elevated markers of hypoxia^23,24^.

GLN values in GLRLM features were also correlated with recurrence-free survival. GLN measures the similarity of grey level intensity values in the image, where a lower GLN value correlates with a greater similarity in intensity values. Therefore, we conclude that heterogeneity is higher in the group with GLN values greater than the median value, and that recurrence-free survival is worse than expected in this group. The GLCM value is determined only on the basis of the relationships among pixel values. Regardless of the position, heterogeneity increases as the number of pixels showing a large difference from adjacent pixels increases. However, in comparison with the GLCM, the GLRLM value is less dependent on the distribution range of pixel values in the image because the pixel values as well as the lengths of the same pixel values are considered together for GLRLM assessments^25^.

Most studies reporting texture analysis of tumour heterogeneity used histogram analysis and GLCM to obtain results. Although GLCM features demonstrate better performance than other texture types, the GLRLM features performed better in the optimal subset. GLCM is suitable for analysing changes in local heterogeneity because it analyses texture changes through the relationship between neighbouring pixels. In contrast, GLRLM is more sensitive for analysing changes in regional heterogeneity because it analyses texture changes through the entire length of the run.

Several studies have reported assessments of tumour heterogeneity by GLRLM texture analysis^26^. For example, Ho *et al*. used GLRLM textural analysis and reported that the heterogeneity of intratumoural FDG distribution and the early temporal changes in total lesion glycolysis may be important predictors for overall survival in patients with bulky cervical cancer^27^.

One limitation of the present study was the use of ROIs covering only the lesion core in pancreatic tumours rather than the entire tumour area or the largest cross-sectional area. In contrast, other studies performed texture analysis of the entire area^28^. Typically, whole-tumour analysis appears to yield results that are more representative of tumour heterogeneity. However, because of the nature of tumours, proliferation usually occurs in the central part of the lesion. It is true that proliferation also occurs at the border of the tumour; however, the development of a necrotic core in cancer patients is correlated with increased tumour size, high-grade disease, and poor prognosis due to the emergence of chemoresistance and metastases^29^. This suggests that the core of the tumour is adequate for predicting heterogeneity.

The second limitation is that this analysis did not reflect genetic heterogeneity. Biologically, to confirm tumour heterogeneity, it is essential to identify genetic heterogeneity. Without also performing genetic analysis, it is difficult to confirm that texture analysis has completely deduced the histological heterogeneity. Previous studies did not correlate pancreatic cancers with molecular markers or investigate subtypes of pancreatic cancers. However, we plan to correlate this texture analysis with genetic heterogeneity and perform genetic analysis using a prospective study design.

Inferring tumour heterogeneity through texture analysis, a radiomics approach, has several advantages and disadvantages. We have identified a tool for prediction of the postoperative oncologic outcome and prognosis through preoperative abdominal CT scans in aggressive cancers such as pancreatic cancer.

No previous studies have reported resectable pancreatic cancers with the same adjuvant treatments, and the present study had a larger sample size than previous studies. Our study has great significance as we performed the same texture analysis with a different method^30,31^.

Despite the fact that this study used retrospective data, our result may indicate an important predictor of prognosis, since we used the preoperative CT scan of resectable pancreatic cancer patients.

In conclusion, the findings obtained with texture analysis show clinical significance in predicting the survival and prognosis of pancreatic cancer patients.

## Methods

### Patients

We included 230 consecutive patients who underwent curative-intent surgical resection for pancreatic cancer at Gangnam Severance Hospital between 2001–2017. Preoperative contrast-enhanced CT data of 116 patients were available for texture analysis. Patients who had undergone neoadjuvant concurrent chemoradiotherapy were excluded from this analysis. The study protocol was approved by the institutional review board at Gangnam Severance Hospital, Yonsei University of Korea (3-2016-0338). The study complied with the Declaration of Helsinki. Informed consent was obtained from all participants.

### CT protocol

For the 116 patients, preoperative dynamic CT was performed using one of two scanners: a 16-slice multidetector CT (MDCT) scanner (Somatom Sensation 16; Siemens Medical Solutions, Erlangen, Germany) or a 64-slice MDCT scanner (Somatom Sensation 64; Siemens Medical Solutions, Erlangen, Germany). All patients were instructed to fast for at least 5 hours before undergoing CT examinations. Each patient was administered 150 mL of a nonionic contrast material (Ultravist 300; Schering AG, Berlin, Germany) intravenously by means of a power injector (EnVision CT; Medrad, Pittsburgh, Pa) at a rate of 3 mL/second.

The CT images were acquired in the craniocaudal direction with the following parameters: detector collimation, 16 × 0.75 mm; table feed, 12 mm per rotation; section width, 3 mm; reconstruction increment, 3 mm with 3-mm-thick sections; pitch, 1.2; tube voltage, 120 kVp; and tube current, 160 mAs. Precontrast scanning (i.e., the first pass) was performed first, followed by contrast-enhanced CT. In order to determine the time of peak aortic enhancement, a bolus injection of 20 mL of contrast material was administered, and sequential dynamic sections were acquired every 2 seconds, starting from the hepatic hilum. Based on the findings of a previous study on multidetector row helical CT, we calculated the start time for the arterial phase by adding 15 seconds to the time of peak aortic enhancement calculated at the hepatic hilum. The resultant average start time for the arterial phase was 34 seconds (range, 30 ± 38 seconds). The equilibrium phase scan was acquired at 3 minutes after the start of the contrast material injection^32^.

### Data analysis

**Figure 2:**
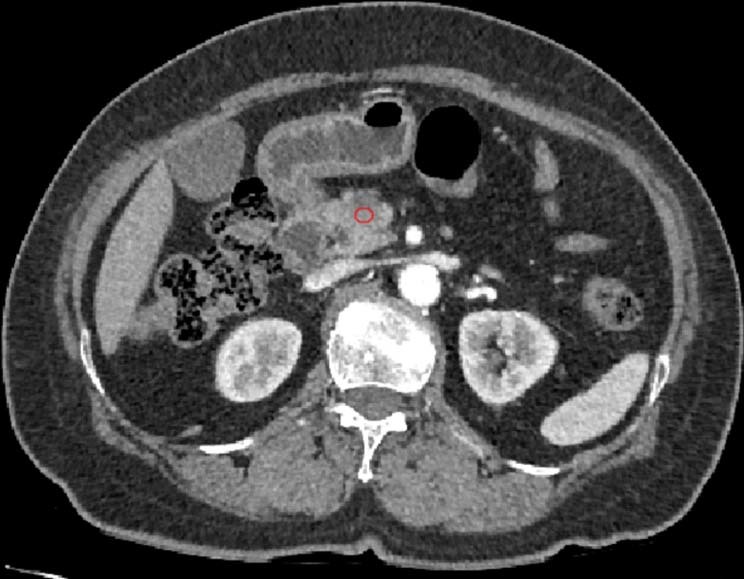
ROI processing in preoperative CT scan.

For each primary cancer site, a region of interest (ROI) was drawn on all slices with a visible tumour on the AP CT scans using the GCUBME-ROI Tool. This tool is a program created independently for this study. The ROIs were reviewed by a radiologist blinded to the patient outcomes. The circular ROI was placed within the central region of the tumour and the normal parenchyma in the pancreas without including the pancreatic and bile ducts and vessels. All ROIs had the same diameter (4 mm) (Fig. 2).

### Texture analysis

For texture analysis, pixel values in the ROIs were transformed into the GLRLM matrix for each CT image. GLRLM provides the size of homogeneous runs for each grey level along a specific linear direction, which is defined by four different directions in the 2D GLRLM, i.e., 0°, 45°, 90°, and 135°. In the GLRLM matrix, the rows are represented by grey values, and the columns are represented by the number of same adjacent pixels. The grey-level non-uniformity (GLN) features were calculated from the GLRLM matrix for the four directions using Eq. (1):

1 $$\mathop{\sum }\limits_{i=0}^{G-1}\,{(\mathop{\sum }\limits_{j=0}^{R-1}{P}_{i,j})}^{2}$$

GLN measures the distribution of runs along the grey levels. The GLN feature value is low when the runs are equally distributed along grey levels. Therefore, a low GLN feature value indicates high similarity of intensity values. In the GLRLM matrix, the rows are represented by grey values, and the columns are expressed by the same number of adjacent pixels^25,33^. The number of occurrences wherein the grey value of each of the pixels is the same as the grey value of the neighbouring pixels in a given direction and distance is represented by a matrix.

### Statistical analysis

All statistical analyses were performed using SPSS software, version 20.0 (SPSS Inc., Chicago, IL). Categorical variables were evaluated using chi-square or Fisher’s exact tests. Survival curves were plotted using the Kaplan–Meier method, and intergroup differences in survival time were assessed with the log-rank test. Recurrence-free survival was defined as the interval between the date of surgery and the date of recurrence. The Cox proportional hazards regression method was used to determine independent prognostic factors. A p-value lower than 0.05 was considered statistically significant.
